# Supplementary material for: Two Consecutive Prolines in the Fusion Peptide of Murine β-Coronavirus Spike Protein Predominantly Determine Fusogenicity and May Be Essential but Not Sufficient to Cause Demyelination
Source: Viruses. 2022 Apr 17;14(4):834. doi: 10.3390/v14040834 (PMC9031231; doi:10.3390/v14040834)
Supplement: Supplementary file 1 [file viruses-14-00834-s001.zip › viruses-1663848-supplementary.pdf]

viruses

MDPI

Supplementary Material

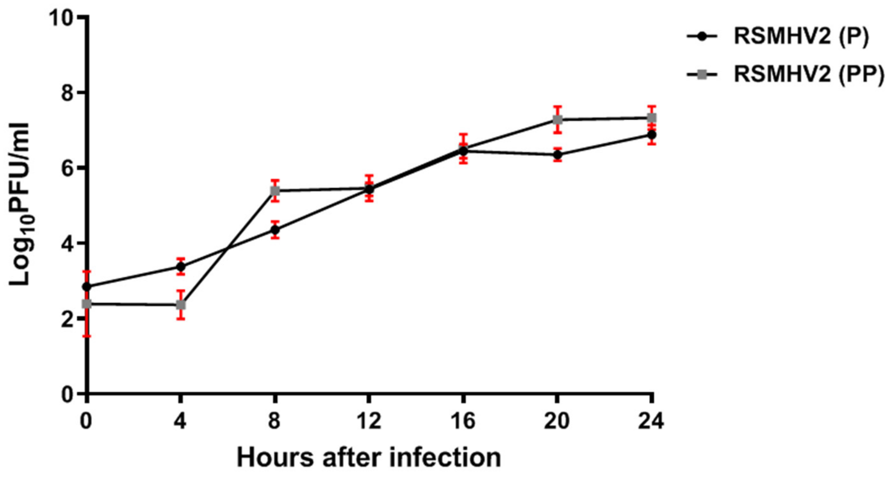

Figure S1. Growth kinetics of RSMHV2 (P) and RSMHV2 (PP): Confluent monolayer of L2 cells were infected with RSMHV2 (P) and RSMHV2 (PP) at MOI of 1. At times 0, 4, 8, 12, 16, 20, and 24 h p.i., the viral titers were determined by routine plaque assay and the Log_{10} PFU/mL was plotted in a graph. There were no differences in the replication kinetics between RSMHV2 (P) and RSMHV2 (PP) in the L2 cell line. Both showed a consistent and similar replication rate.

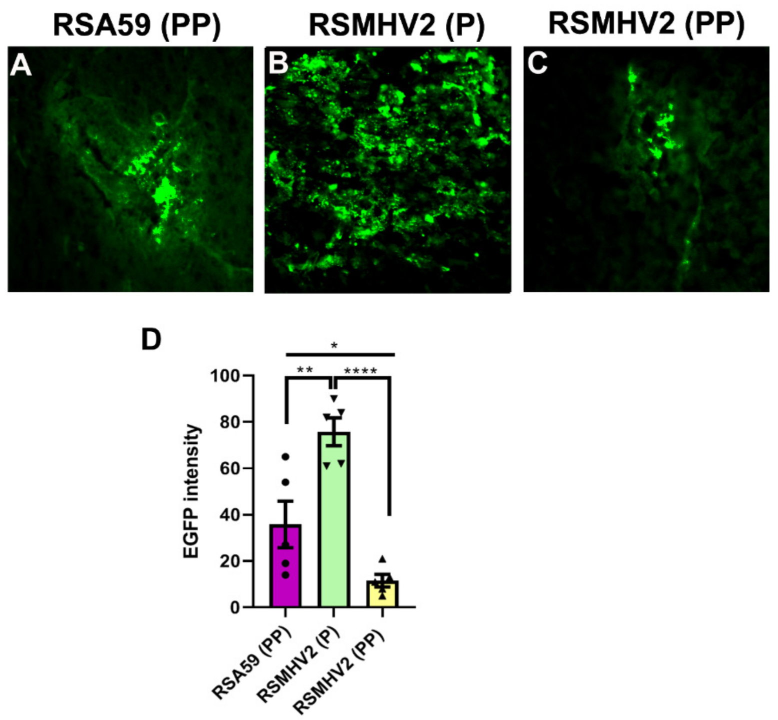

Figure S2. Viral antigen spread in brain parenchyma of RSA59 (PP), RSMHV2 (P), and RSMHV2 (PP) infected at 200 PFU at day 5 p.i. RSA59 (PP), RSMHV2 (P), and RSMHV2 (PP) were inoculated at 200 PFU and day 5 p.i. brain tissues were harvested, and cryosections were processed for epifluorescence microscopy to visualize EGFP (viral antigen). RSA59 (PP) (A), RSMHV2 (P) (B), and RSMHV2 (PP) (C) at 200 PFU showed differential but comparable viral antigen infectivity and spread. The intensity of EGFP was quantified and plotted in a scatter-bar diagram (D). Unpaired Student's t-test calculated the significance level. N=3 mice per virus infection. *$p<0.05$, **, $p<0.001$; ****, $p< 0.0001$.
